# Clinch-Bonding Process for Ultra-High-Strength Steel and A5052 Aluminum Alloy Sheets

**DOI:** 10.3390/ma18153556

**Published:** 2025-07-29

**Authors:** Yohei Abe, Yu Tatara, Takahiro Hosokawa, Ryoto Yamauchi

**Affiliations:** Department of Mechanical Engineering, Toyohashi University of Technology, 1-1 Hibarigaoka, Tempaku, Toyohashi 441-8580, Japan

**Keywords:** joining, mechanical clinching, adhesive, material flow, joining conditions, frictional effect, fine particles, sheets

## Abstract

Initially, the effects of sheet combinations for joining two sheets, including 780 MPa steel and A5052 aluminum alloy sheets, on the joined cross-sectional shapes of the sheets in a clinch-bonding process and the tension-shear load of joined sheets were investigated. The effect of an adhesive on the amounts of the interlock and the minimum thickness in the upper sheet was not large, whereas the effect of the sheet combination was observed. Subsequently, for joining the upper 980 MPa ultra-high-strength steel and lower aluminum alloy sheets in the clinch-bonding process, the effects of the die shape, punch velocity, and sheet holding force on the joinability were investigated. As a result, defect-free conditions were narrowly constrained. Finally, a method that involved controlling material flow using an adhesive with fine particles to increase friction between the sheets was introduced. The upper 980 MPa steel and lower aluminum alloy sheets were successfully joined using this approach.

## 1. Introduction

In recent years, the automotive industry has focused on reducing energy consumption and CO_2_ emissions. For reducing energy consumption and CO_2_ emissions, weight reduction of various automotive components is an expected method. Weight reduction of automotive bodies is expected to improve fuel efficiency, and body structure changes using lightweight high-strength composites such as carbon fiber-reinforced plastics, light metals like aluminum alloy and magnesium alloy, and high-strength steels such as ultra-high-strength steel sheets are being considered. Among these, high-strength steels and aluminum alloys are the most widely used due to their cost-effectiveness and availability [1]. Emissions differ greatly depending on the amount of electricity required to refine new and recycled aluminum ingots, and efforts to promote recycled ingots with lower CO_2_ emissions are expanding [2].

In the bodies of higher-priced automobiles, many aluminum alloy parts are used. Considering the cost of aluminum alloys and their properties as materials for structure, large one-piece castings have been introduced to significantly reduce the number of parts [3]. However, partial application based on the functional requirements of each part is also effective in lower-priced automobiles. Partial components consist mainly of 5000-series and 6000-series alloys formed by stamping and extrusion because the 5000-series alloys have an excellent strength-to-weight ratio, high formability, and high recyclability, while the 6000-series alloys offer versatility, good weldability, heat treatability, and excellent plasticity [4].

When using these aluminum alloy parts, multi-material joining, such as the joining of steel and aluminum, has become an important technical issue. For butt joints of steel and aluminum, joining processes using heat, such as friction stir welding [5], laser welding [6], and TIG welding-brazing [7], have been developed. For lap joints, not only resistance spot welding using heating [8] but also various mechanical joining methods have been developed [9]. Mechanical joining methods include conventional bolt-and-nut joints, self-pierce rivets [10], flow drilling screws [11], and mechanical clinching [12], among others. Adhesive bonding is an alternative method and has attracted attention as a method that makes it easy to join dissimilar materials [13].

There are several advantages to the lap joints using adhesive bonding. Joint stiffness tends to be larger than that of point joining such as spot welds and mechanical fasteners because of a larger adhesive contact area. In addition, not only does the adhesive layer with a larger area acts as a seal to protect the joint from moisture and debris ingress, but it also prevents contact of dissimilar sheets, which could otherwise lead to galvanic corrosion [14,15]. Although heat-curing epoxy-based adhesives are used in car bodies due to high joint strength [16], another joining method is required to hold the joined parts in place while curing in a furnace. Mechanical joining methods are suitable for joining aluminum alloy and steel sheets using epoxy-based adhesives.

The selection of a mechanical joining method combined with the adhesive depends on the joint strength and the joining cost. When high joint strength is required despite a degraded adhesive layer, high-strength joint methods such as self-pierce riveting and flow drilling screwing are preferable. Conversely, if adequate degradation prevention is achieved, mechanical clinching with the adhesive, clinch-bonding, would be a suitable option for reducing joining costs. Although clinch-bonding provides high joint strength [17,18] and high joint energy absorption [18,19], the gap between sheets in the adhesive layer can be significant due to sheet deformation during self-pierce riveting with the adhesive [20]. In clinch-bonding involving high-strength steel and aluminum alloy sheets, the joint strength has been observed to be affected [21].

In clinching, frictional conditions affect the material flow of the sheets. These conditions include coating layers on the top and bottom surfaces of the sheets, as well as lubrication between the sheets and the tools. When clinching hot-dip-coated steel sheets, the deformed shapes were similar to those of sheets with removed coatings, although the coating layers on the sheet surfaces were shaved or scratched in clinching [22]. In clinch-bonding, the adhesive layer between the upper and lower sheets influences the material flow of the sheets. The effect of the friction among the sheets and tools for interlocking in clinched sheet without the adhesive was investigated [23]. It was shown that the interlock in clinch-bonding was reduced compared to that without the adhesive in a finite element simulation considering deformations in the adhesive elements and an experiment [24]. The adhesive dynamic behavior in the clinch-bonding process of aluminum alloy and 780 MPa high-strength steel sheets was investigated. Subsequently, the flow-out of the adhesive from the deforming region and the prediction of fracture were calculated using a proposed finite element simulation [25].

Tension-shear tests are widely employed to assess the mechanical integrity of clinched joints [26], irrespective of the presence of adhesives. Observed failure modes typically include upper sheet fracture at the minimum wall thickness section, disconnecting from the interlocking region, or a combination of both [27]. The joint load in tension-shear tests has been predicted based on the separation form of joints without the adhesive [28]. The strength of clinch-bonded joints has also been evaluated using damage models for both single [29] and double joints [21]. However, even for the same sheet combination, strength is often assessed by joint load alone, and consistent evaluation using joint efficiency—based on the load of the lower-strength sheet being joined—is important.

As shown in the previous work [30], a two-sheet combination of aluminum alloy A5052 and 780 MPa high-strength steel or 980 MPa ultra-high-strength steel sheets represented a critical condition without the adhesive using mechanical clinching through flow control of sheets with a different die shape. In clinch-bonding for these combinations, not only the influence of the adhesive but also the large strength difference between the upper and lower sheets with low ductility must be considered. Therefore, in order to extend the applicable joining range, it is important to clarify the material flow of the sheets with the adhesive in these combinations.

In this study, the effects of sheet combinations on the cross-sectional shapes of the joined sheets in the clinch-bonding process for joining two sheets, including 780 MPa steel and A5052 aluminum alloy sheets, and the tension-shear load of the joined sheets were investigated. The tension-shear loads of the joints were then summarized as joint efficiency and compared. For joining the upper ultra-high-strength steel and lower aluminum alloy sheets, the effects of the die shape, punch velocity, and sheet holding force on joinability were investigated. Finally, an adhesive with fine particles was introduced to join the upper ultra-high-strength steel and lower aluminum alloy sheets by controlling material flow.

## 2. Clinch-Bonding Process for 780 MPa High-Strength Steel and Aluminum Alloy Sheets

### 2.1. Sheet Materials, Clinching Methods, and Tension-Shear Test Conditions

**Figure 1:**
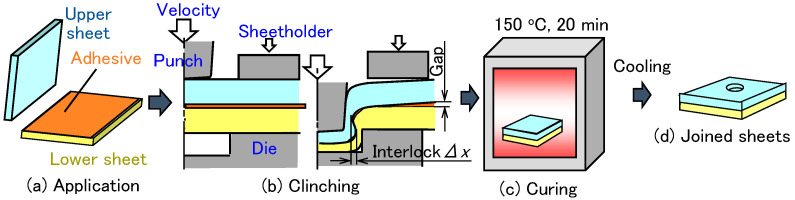
Clinch-bonding process with epoxy-based adhesive. (**a**) Application, (**b**) clinching, (**c**) curing, and (**d**) joined sheets.

A clinch-bonding process is shown in Figure 1. In the clinching process for automobile body panels, an epoxy-based adhesive is assumed. First, the adhesive is applied to the bonding surface of the top side of the lower sheet. Next, the sheets are placed on the die. The sheets are then pressed by a punch, generating an interlock between the upper and lower sheets to join them. After clinching, the adhesive in the joint is cured by heating in a furnace. Once heated and cured, the joint is cooled in air. Finally, the sheets are fully joined. Although the deformation behavior of the sheets during curing is important, it is essential not only to generate an interlock without defects but also to maintain a small gap in the flange portion between the sheets to ensure an effective adhesive layer, which contributes to achieving high joint strength.

In this study, a 1.2 mm thick 780 MPa steel sheet was selected as the high-strength steel sheet, and a 1.5 mm thick aluminum alloy A5052-H34 sheet was selected. The mechanical properties of the sheets, determined by a uniaxial tensile test in accordance with the standard [31] using a universal testing machine (Autograph AGS-J, Shimadzu Co., Kyoto, Japan), are summarized in Table 1. The tensile strength of the steel sheet is three times that of the aluminum alloy sheet.

**Figure 2:**
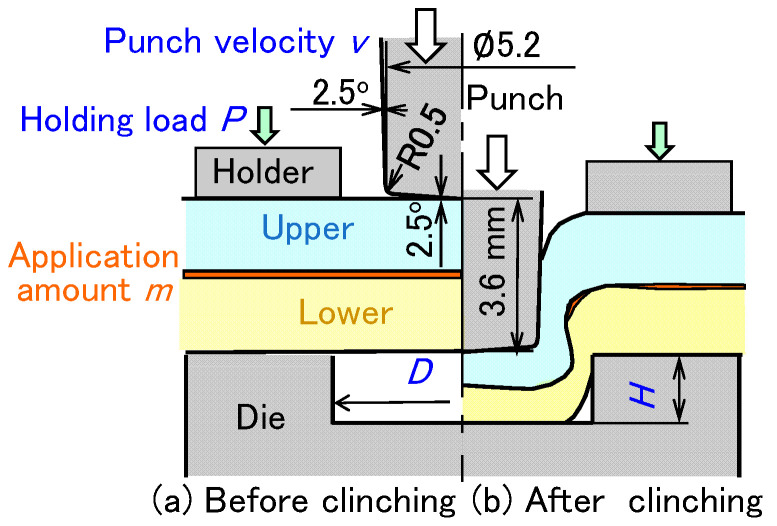
Clinching conditions with the adhesive. (**a**) Before clinching and (**b**) after clinching.

The clinching conditions with the adhesive are shown in Figure 2. A 5.2 mm punch diameter was used with a different die shape and a punch stroke for each sheet combination. The sheet combinations and clinching conditions for clinch-bonding are shown in Table 2. The die diameter *D* (mm), the die depth *h* (mm), and the final punch stroke *s* (mm) were specified for each sheet combination. The conditions were determined by an experiment without the adhesive. The parameters, except for the die diameter *D* (mm), the die depth h, and final punch stroke *s* (mm), were the application amount *m* (g/m^2^), punch velocity *v* (mm/s), and holding load *P* (kN). In this chapter, *m* = 150 g/m^2^, *v* = 26 mm/s, and *P* = 5 kN were used. The epoxy-based adhesive was EP138 (CEMEDINE Co., Ltd., Tokyo, Japan). The viscosity of the adhesive at 20 °C was 180 Pa·s, measured by a viscometer VT-06 (RION Co., Ltd., Tokyo, Japan). The joint tests were performed using an 800 kN CNC servo press (SDE-8018, AMADA PRESS SYSTEM Co., Ltd., Isehara, Japan).

**Figure 3:**
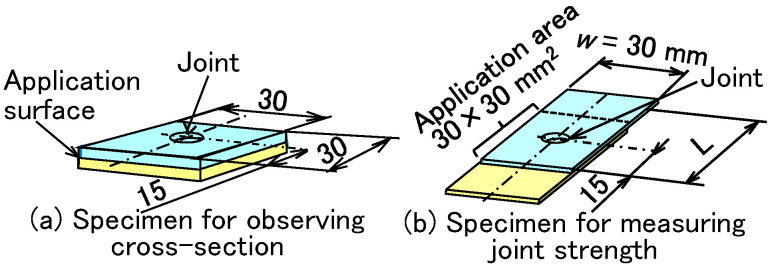
Specimens for joining steel and aluminum alloy sheets. (**a**) Specimen for observing cross-section, and (**b**) specimen for measuring joint strength.

The specimens for joining two sheets are shown in Figure 3. Two types of specimens were used. One was a specimen for observing the cross section of the sheets with a digital microscope VHX-7000 (Keyence, Osaka, Japan) as shown in Figure 3a. The other was a specimen for measuring joint strength in a tension-shear test in Figure 3b. In both specimens, the adhesive was applied to the overlapped surface between the upper and lower sheets. The application area was 30 × 30 mm^2^. In the tension-shear test, the specimen with bracing sheets was pulled at a rate of 10 mm/min using a universal testing machine (Autograph AGS-J, Shimadzu Co., Kyoto, Japan). The maximum load and fracture behaviors of sheets were investigated.

### 2.2. Experimental Results of Joined Sheets

**Figure 4:**
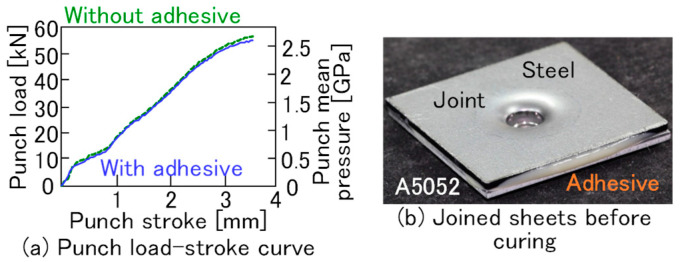
Punch load-stroke curves with and without the adhesive and joined sheets before curing. (**a**) Punch load-stroke curve, and (**b**) joined sheets before curing.

The punch load-stroke curves and the joined sheets before curing are shown in Figure 4. In the punch load-stroke curve, the mean punch pressure, which is punch load divided by the cross-sectional area of the punch, is presented in Figure 4a. The punch load increased with the increasing stroke. The final mean punch pressure is very high, exceeding 2.5 GPa. The difference between the conditions with and without the adhesive is very small. The overflowing adhesive between the upper and lower sheet edges after clinching was observed and is shown in Figure 4b.

**Figure 5:**
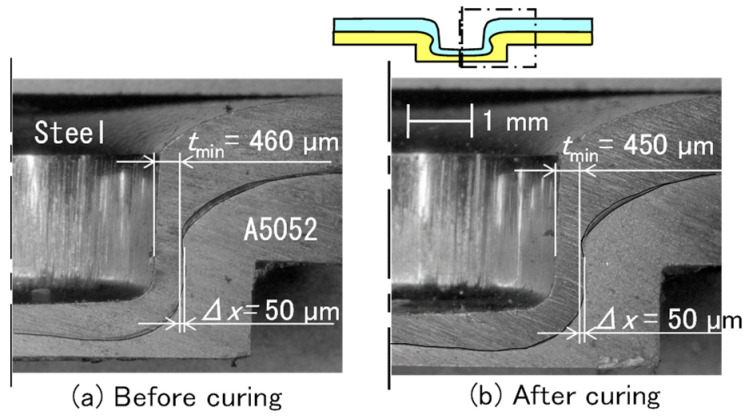
Cross-sectional shapes of joined sheets before and after curing. (**a**) Before curing and (**b**) after curing.

The cross-sectional shapes of the joined sheets before and after curing are shown in Figure 5. The amounts of the interlock and minimum thickness in the upper sheet were the same before and after curing. The difference in the minimum thickness between before and after curing is due to an error in the joining process.

**Figure 6:**
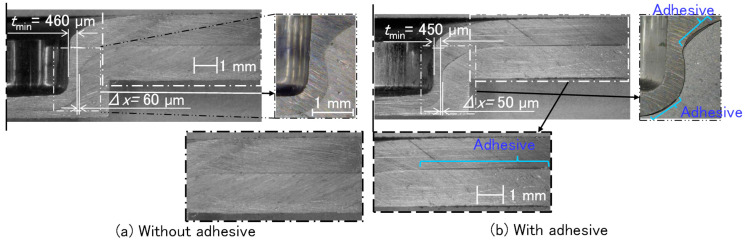
Cross-sectional shapes of joined sheets after curing for 780 MPa steel-A5052 sheets. (**a**) Without the adhesive and (**b**) with the adhesive.

The cross-sectional shapes of joined sheets after curing for the 780 MPa steel-A5052 sheets are shown in Figure 6. The amounts of the interlock with the adhesive were reduced. This tendency caused by the adhesive layer between sheets was also simulated by Etemadi et al. [24]. In the cross-sectional shape without the adhesive, neither the gap nor the adhesive layers between the upper and lower sheets were observed. The adhesive layers in the flange portion and around the minimum thickness in the upper sheet were observed between the upper and lower sheets.

**Figure 7:**
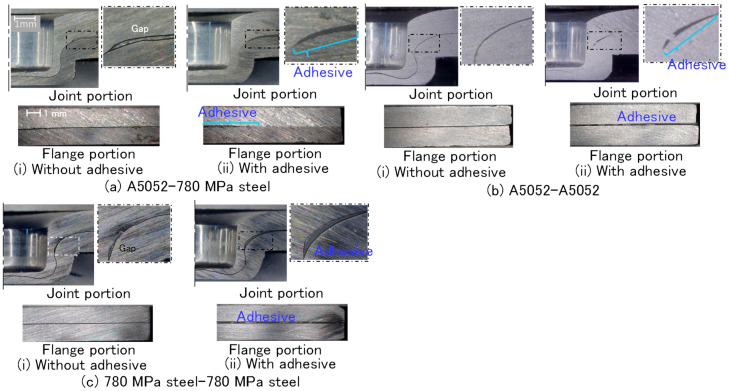
Cross-sectional shapes of joined sheets. (**a**) A5052-780 MPa steel, (**b**) A5052-A5052, and (**c**) 780 MPa steel-780 MPa steel.

The cross-sectional shapes of joined sheets after curing in the other sheet combinations were investigated, and the shapes are shown in Figure 7. In the A5052-780 MPa steel sheets without the adhesive, the gap between the upper and lower sheets above the die surface was observed. The gap was filled with the adhesive in the cross section with the adhesive, whereas the adhesive layer at the edge of the flange portion was not observed. In both the A5052 sheets without the adhesive, the gap between the upper and lower sheets was not observed. The adhesive layer in the cross section with the adhesive was observed from the outside of the minimum wall thickness to the flange edge of the sheets. In both the 780 MPa steel sheets, the behavior of the adhesive layer was similar to that in the A5052 sheets.

**Figure 8:**
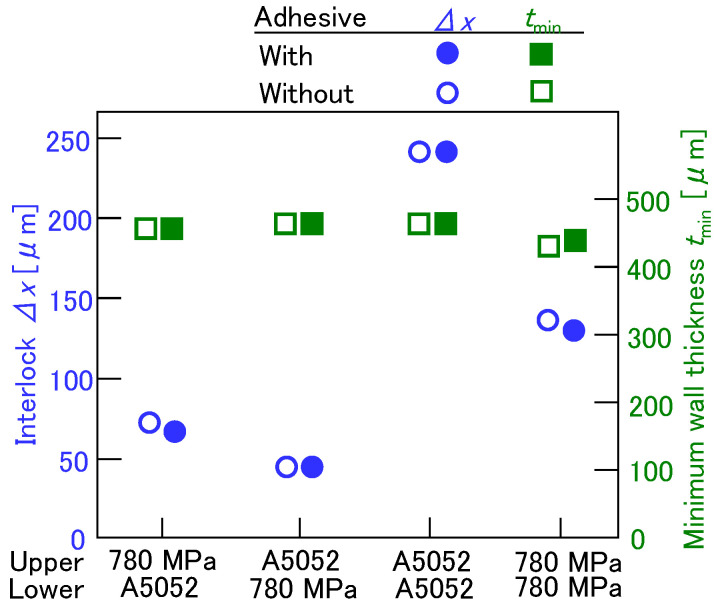
Effect of adhesive on interlock and minimum wall thickness.

The effect of the adhesive on the interlock and the minimum wall thickness is shown in Figure 8. Although the effect of the sheet combination on the interlock was observed, the effect of the adhesive on the interlock and the minimum wall thickness was small, despite the interlocks with the adhesive being smaller. Thus, the effect of the friction between the upper and the lower sheets was not large under these conditions.

### 2.3. Experimental Results of Joint Strength

**Figure 9:**
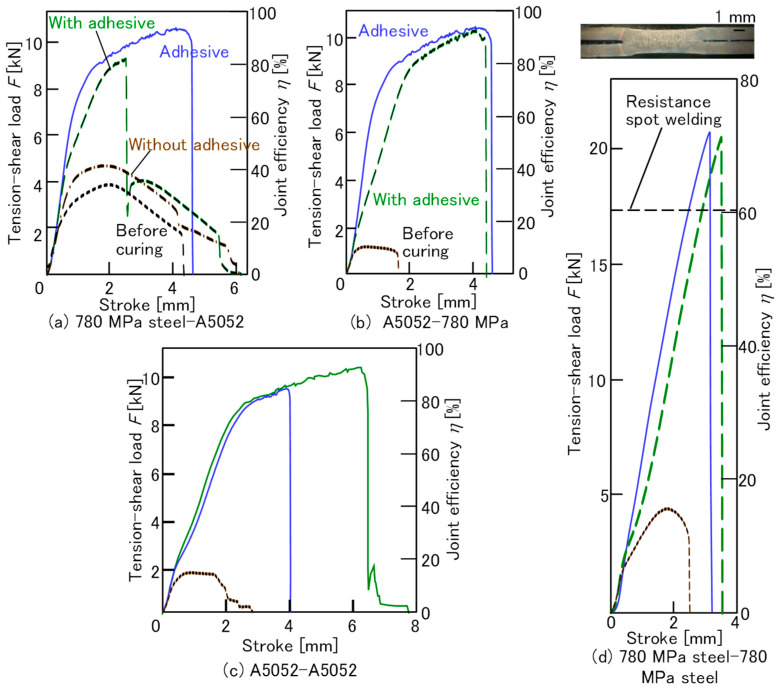
Tension-shear load-stroke curves. (**a**) 780 MPa steel—A5052, (**b**) A5052—780 MPa, (**c**) A5052—A5052, and (**d**) 780 MPa steel—780 MPa steel.

The tension-shear load *F* (kN)-stroke curves are shown in Figure 9. In the tension-shear load-stroke curves, the joint efficiency *η*, defined as the load divided by the product of the cross-sectional area and the tensile strength of the lower strength sheet $\sigma_{B}$ (MPa), is indicated in the figures.

(1) $$\eta =\frac{F}{\sigma_{B}\;t\;w}$$

where *t* and w are the thicknesses of the lower sheet and the width of the specimen for measuring joint strength in Figure 3b, respectively. ‘With adhesive’, ‘Adhesive’, and ‘Without adhesive’ in the figure indicate the conditions of clinching with the adhesive, using the adhesive only, and clinching without the adhesive, respectively. In all sheet combinations, the maximum loads without the adhesive were lower than those with the adhesive. In both the 780 MPa steel sheets, the maximum loads in each condition were higher than those of the sheet combination, including the A5052 sheet, because of the high strength of the steel sheet. In the 780 MPa steel-A5052 sheets, the load before curing was the lowest because of the low friction of the adhesive layer between the upper and lower sheets. The adhesive load was the highest, and then the load of clinching with the adhesive was the second highest. The load with the adhesive suddenly dropped at around 2.4 mm in stroke, and the behavior after the drop was similar to the load without the adhesive. Similar fracture characteristics were observed in reference [18]. In the other sheet combinations, the order of the high load condition was the same as the order for the 780 MPa steel—A5052 sheets. Although a small load at around 6.6 mm in stroke with the adhesive after the drop in both A5052 sheets was observed, the load with the adhesive suddenly dropped to 0 kN at a certain stroke.

**Figure 10:**
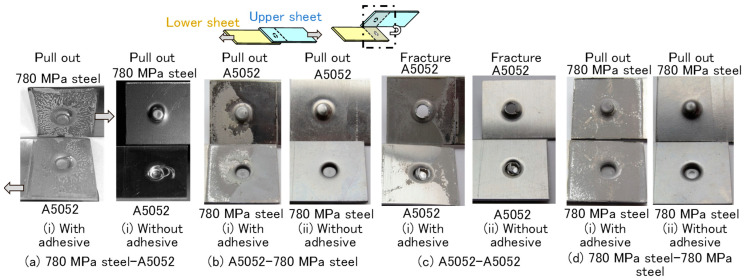
Specimens after tension-shear test. (**a**) 780 MPa steel—A5052, (**b**) A5052—780 MPa, (**c**) A5052—A5052, and (**d**) 780 MPa steel—780 MPa steel.

The specimens after the tension-shear test are shown in Figure 10. In both A5052 sheets with the adhesive, peeling adhesive and a fracture around the minimum wall thickness of the upper sheet were observed. The small load at around 6.6 mm in stroke in Figure 9c seems to be caused by this fracture. In other sheet combinations with the adhesive, the peeling adhesive and the pulling out of the interlock were observed. The fracture behaviors with and without the adhesive were similar in each sheet combination.

**Figure 11:**
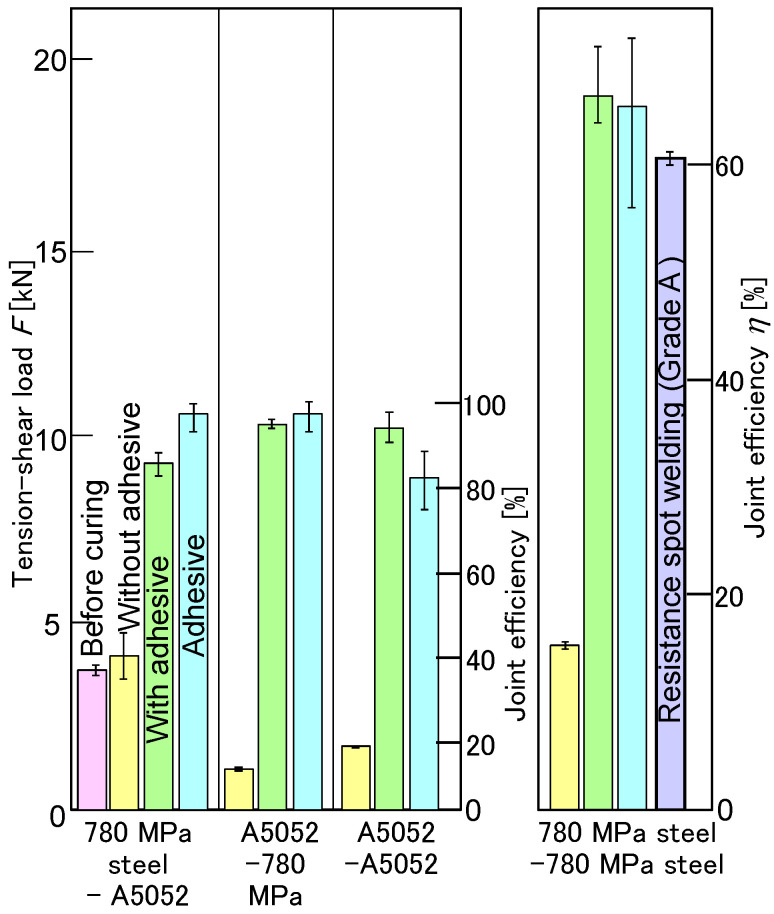
Maximum tension-shear loads.

The tension-shear loads are summarized in Figure 11. The maximum load, including A5052 sheet with the adhesive, was over 80% joint efficiency, although the joint efficiency of the clinching without the adhesive was less than 15%. The maximum load for both 780 MPa sheets with the adhesive was about 65% in joint efficiency, which was the same joint efficiency as the adhesive joint. The clinching process with the adhesive is effective for improving joint efficiency.

## 3. Clinch-Bonding for Upper 980 MPa Ultra-High-Strength Steel and Lower Aluminum Alloy Sheets

### 3.1. Sheet Materials and Clinching Methods

In the sheet combination of the upper high-strength steel and lower aluminum alloy sheets, the fracture in the upper sheet during joining tends to occur due to the concentration of the deformation in the low ductility of the high-strength steel. In the clinching with the adhesive, the tendency may be accelerated by the adhesive between the upper and lower sheets. In this chapter, the effect of adhesive on the upper ultra-high-strength steel sheet having a high strength and a low ductility was investigated.

**Figure 12:**
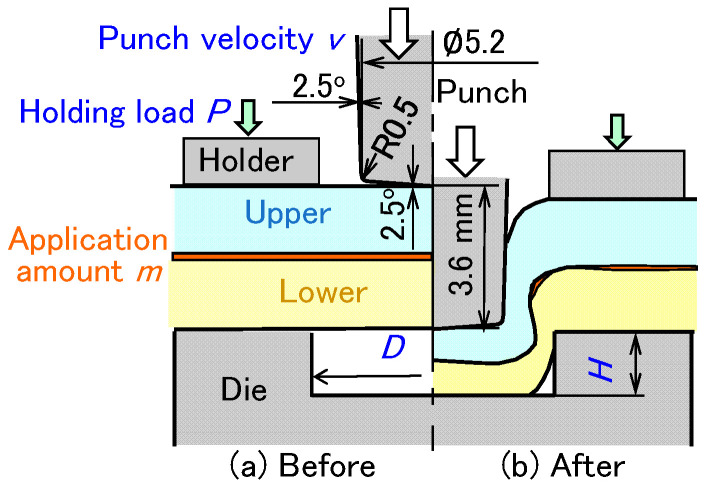
Conditions of clinch-bonding for upper ultra-high-strength steel sheets. (**a**) Before clinching and (**b**) after clinching.

The conditions of clinch-bonding for the upper ultra-high-strength steel sheet are shown in Figure 12. First, the effects of the die diameter *D* = 8 to 9 mm and die depth *h* = 1.4 to 1.6 mm under *m* = 150 g/m^2^, *v* = 26 mm/s, and *P* = 5 kN were investigated. Then, the effects of the punch velocity *v* (mm/s) and holding load *P* (kN) under *m* = 150 g/m^2^ and selected die shape were investigated. Finally, the effect of the application amount of adhesive was observed.

The material properties of the steel sheets are shown in Table 3. The 980 MPa ultra-high-strength steel sheet was used. Not only the 780 MPa steel sheet but also a lower-strength 590 MPa steel sheet was used for comparison.

### 3.2. Methods and Conditions of Finite Element Simulation

**Figure 13:**
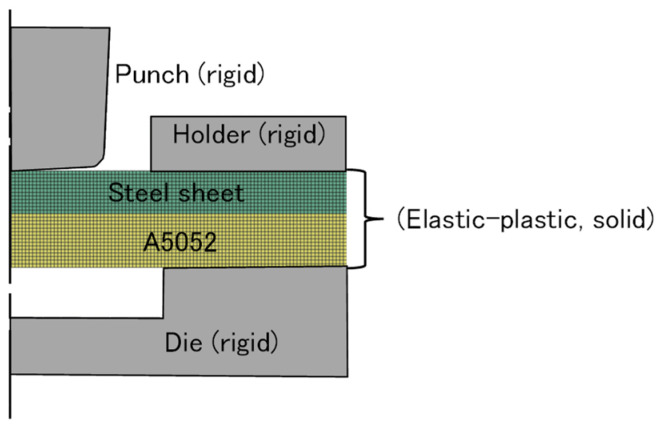
The finite element model for clinching.

To understand the effect of friction between the upper and lower sheets, the finite element simulation was used. The finite element model for clinching is shown in Figure 13. The solver used was LS-DYNA Version R15 (JSOL CORPORATION, Tokyo, Japan) with a dynamic explicit method. The sheets were assumed as an elastic-plastic material with a flow stress as follows:

(2) $$\sigma =K\varepsilon^{n}$$

where *K* and *n* are material constant and work-hardening exponent, respectively, and the values are presented in Table 4. Each four-node quadrilateral element within the sheets measures 0.1 mm. Due to significant deformation, the sheets are periodically remeshed. The tools were assumed to be rigid in an axisymmetric model. The Coulomb friction was applied to all interfaces. A coefficient of friction of 0.2 was assumed between the tools and sheets; the coefficient of friction between the upper and lower sheets was treated as a parameter. The deformed shapes of the sheets were visualized using Jvision Version 3.10.0 (JSOL CORPORATION, Tokyo, Japan).

### 3.3. Experimental and Simulated Results of Deforming Behaviors of Sheets

**Figure 14:**
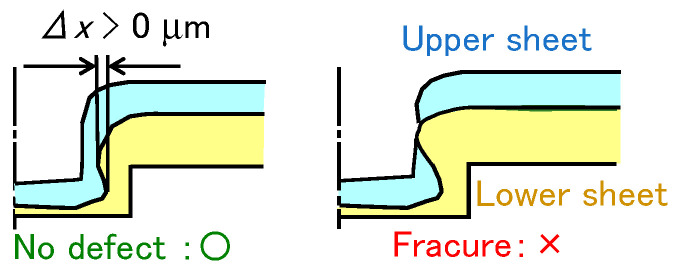
Classification for clinched sheets.

The classification of clinched sheets is shown in Figure 14. In the experiment, not only were joined sheets without defects observed, but also a fractured upper sheet.

**Figure 15:**
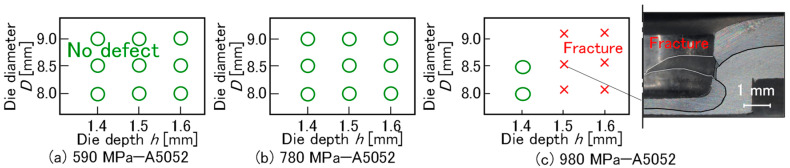
The effect of die geometry on joinability for *P* = 5 kN, *v* = 26 mm/s, and *m* = 150 g/m^2^. (**a**) 590 MPa—A5052, (**b**) 780 MPa—A5052, and (**c**) 980 MPa—A5052.

The effect of die geometry on joinability for *P* = 5 kN, *v* = 26 mm/s, and *m* = 150 g/m^2^ is shown in Figure 15. In the 590 MPa and 780 MPa steel sheets, joining was successful without defects under all conditions. In the 980 MPa steel sheet, the conditions in the sheets without defects were limited. Fractures tended to occur due to the insufficient ductility of the sheet in the deep die. As the die depth decreased, the concentration of deformation around the corner of the punch was reduced [28].

**Figure 16:**
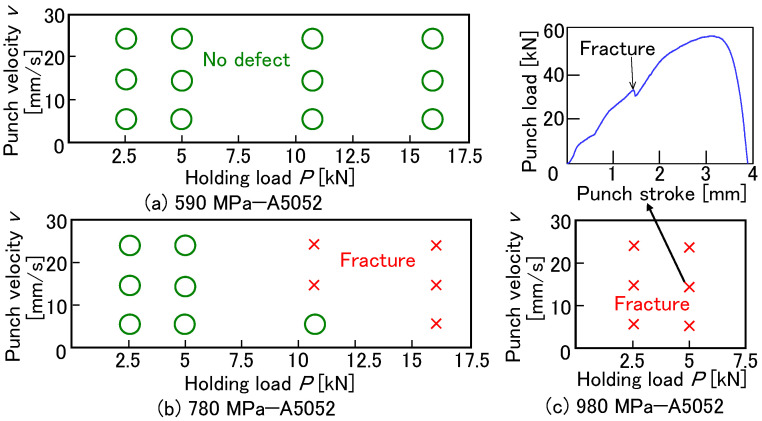
Effects of punch velocity and holding load on joinability for *D* = 8.5 mm, *h* = 1.5 mm, and *m* = 150 g/m^2^. (**a**) 590 MPa—A5052, (**b**) 780 MPa—A5052, and (**c**) 980MPa—A5052.

The effect of the adhesive between the upper and lower sheets decreases with a low punch velocity and a low holding load, i.e., the adhesive tends to flow out from the interface during a long forming time under a relaxed constraint. It should be noted that a certain minimum holding load is required for joining and that a long forming time reduces productivity. The effects of the punch velocity and holding load on joinability for *D* = 8.5 mm, *h* = 1.5 mm, and *m* = 150 g/m^2^ are shown in Figure 16. In the 590 MPa steel sheet, joining was successful without defects under all conditions. In the 780 MPa steel sheet, the conditions in the sheets without defects were limited under a low holding load. In the 980 MPa steel sheet, defect-free conditions were not achieved. Joining the 980 MPa steel sheet and the aluminum alloy proved challenging.

**Figure 17:**
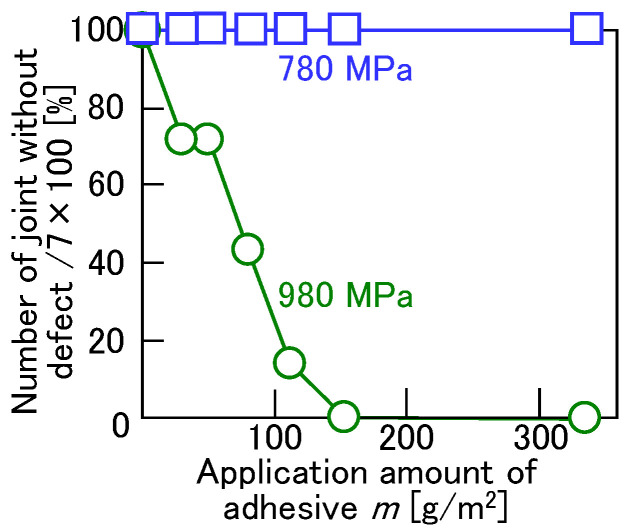
Effects of applied adhesive amount on joinability for *D* = 8.5 mm, *h* = 1.5 mm, *P* = 5 kN, and *v* = 26 mm/s.

The effect of the amount of adhesive was investigated. The effects of the applied adhesive amount on the joinability for *D* = 8.5 mm, *h* = 1.5 mm, *P* = 5 kN, and *v* = 26 mm/s are shown in Figure 17. The joining process was performed seven times for each condition. In the figure, *m* = 0 g/m^2^ represents the condition without the adhesive. Although the 780 MPa steel sheet and A5052 sheets were joined without defects, the ratio without defects decreased with increasing adhesive application in the 980 MPa steel and A5052 sheets. This result suggests that the material flow of the steel sheet was influenced by the frictional effect and the flow stress effect between the sheets due to the adhesive.

**Figure 18:**
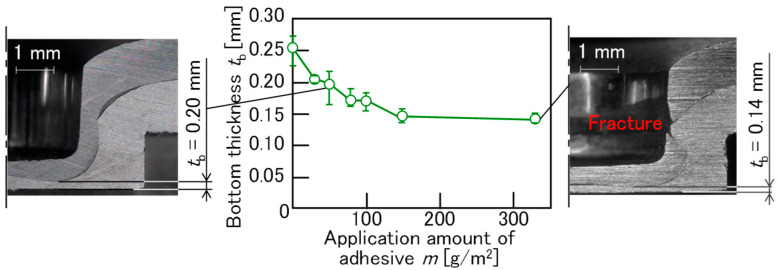
The effect of the application amount of adhesive on material flow for 980 MPa, *D* = 8.5 mm, *h* = 1.5 mm, *P* = 5 kN, and *v* = 26 mm/s.

To investigate the material flow of the sheets, the cross-sectional shape of the sheets after joining was observed. The effect of the applied adhesive amount on the material flow for 980 MPa, *D* = 8.5 mm, *h* = 1.5 mm, *P* = 5 kN, *v* = 26 mm/s, and punch stroke = 3.45 mm is shown in Figure 18. A short punch stroke was applied to clearly differentiate the conditions. The bottom thickness of the A5052 sheet was measured and showed a decrease with the increase in the application amount of adhesive. This indicates that the material flow of the A5052 sheet in the radial direction was increased. It seems that the increased material flow pushed up the steel sheet in a clearance between the punch and die, ultimately causing a fracture in the upper steel sheet.

**Figure 19:**
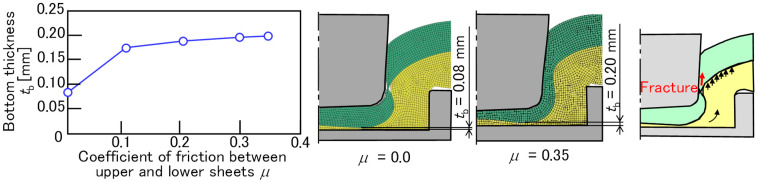
The effect of the coefficient of friction on material flow for 980 MPa, *D* = 8.5 mm, *h* = 1.5 mm, and *P* = 5 kN.

To show the material flow of the sheets, the finite element simulation was performed. Although modeling a finite element simulation that includes adhesive is possible [23], the effect of the coefficient of friction between the upper and lower sheets without the adhesive was investigated. The effect of the coefficient of friction on the material flow for 980 MPa, *D* = 8.5 mm, *h* = 1.5 mm, and *P* = 5 kN is shown in Figure 19. The bottom thickness of the A5052 sheet increases with the increasing coefficient of friction. Because the large amount of adhesive with high viscosity between the sheets is generally assumed to result in a lower coefficient of friction, this trend was consistent with the experimental results. Thus, the material flow of the steel sheet was influenced by the frictional effect of the adhesive between the upper and lower sheets.

**Figure 20:**
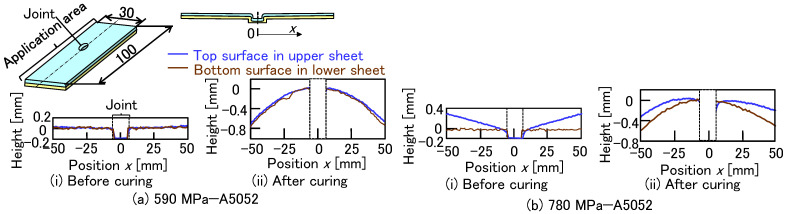
Profiles of the top surface in the upper sheet and the bottom surface in the lower sheet before and after curing. (**a**) 590 MPa—A5052 and (**b**) 780 MPa—A5052.

The profiles of the top surface in the upper sheet and the bottom surface in the lower sheet before and after curing are shown in Figure 20. The sheet combinations without the defects, including the 590 MPa and 780 MPa steel sheets, were investigated. The profiles at the center in the longitudinal direction of the specimen were measured by a laser displacement meters (CD22-15-485M12, OPTEX FA Co., Ltd., Kyoto, Japan). A longer 100 mm specimen was used to clearly observe the trend. In the 590 MPa steel and A5052 sheets before curing, both heights were almost flat and similar. After curing, both heights decreased as the position *x* (mm) increased from the joint center, although both heights were similar. The height change after curing was caused by the deformation of the sheets during heating. In the 780 MPa steel and A5052 sheets before curing, the height of the top surface increased with the increasing position, although the height of the bottom surface remained flat. This tendency was caused not only by the material flow in joining but also by the effect of springback. After curing, both heights decreased with the increasing position. The difference between the top and bottom surfaces in each position remained the same as the difference before curing. Although the gap tendency is not evident in the maximum tension-shear loads shown in Figure 11 using short-length specimens, the loads in long-length specimens may be reduced due to the increased gap between the sheets. In actual components, not only the specimen size but also the constraining conditions may influence the gap tendency.

## 4. Clinch-Bonding for Upper Ultra-High-Strength Steel and Lower Aluminum Alloy Sheets Using Adhesive Containing Fine Particles

### 4.1. Clinching Methods Using Adhesive Containing Fine Particles

In the previous chapter, the material flow of the steel sheet was influenced by the frictional effect of the adhesive between the upper and lower sheets. To reduce the material flow of the steel sheet, methods for increasing the friction between them are required. On the other hand, lubricant oil containing fine ceramic particles has been applied in cup ironing [32] and rod extrusion [33]. These studies reported that the seizure limit increased when using the lubricant, while the forming loads also rose when particles with diameters below one micrometer were employed. The higher forming loads were attributed to the increased frictional force between the material and the die. Consequently, the material flow of the steel sheet may be suppressed due to the enhanced frictional resistance.

**Figure 21:**
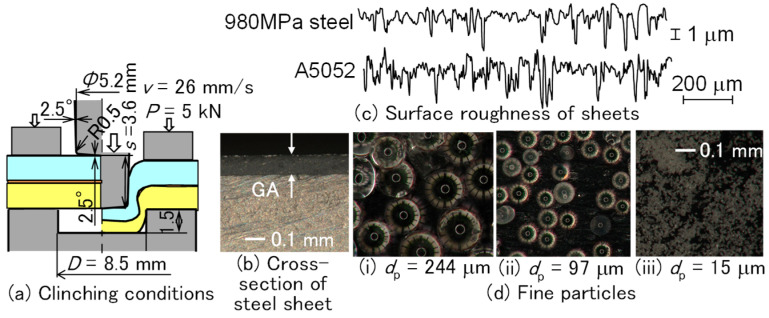
Clinch-bonding conditions and fine particles. (**a**) Clinching conditions, (**b**) cross-section of steel sheet, (**c**) surface roughness of sheets, and (**d**) fine particles.

To join the upper 980 MPa ultra-high-strength steel and lower aluminum alloy sheets using clinching with the adhesive, fine particles to increase friction were added in the adhesive. The fracture of the steel around the sidewall of the punch can be prevented by reducing the material flow of the aluminum alloy sheet, as shown in Figure 19. The clinch-bonding conditions using adhesive with fine particles are shown in Figure 21. Glass beads were selected as fine particle material because of their high hardness, uniform particle size, availability, and low cost. The effects of particle size and percentage content were investigated experimentally. The upper and lower sheets were the 980 MPa ultra-high-strength steel and A5052, respectively. Galvanized alloy layers were present on both surfaces of the steel, as shown in Figure 21b. The glass beads were added to the adhesive. The mean diameter *d*_p_ (μm) of the glass beads in Figure 21c is 15, 97, and 244 μm. The percentage content *c* (mass%) ranged from 0 to 50 mass%.

### 4.2. Experimental Results Using Adhesive Containing Fine Particles

**Figure 22:**
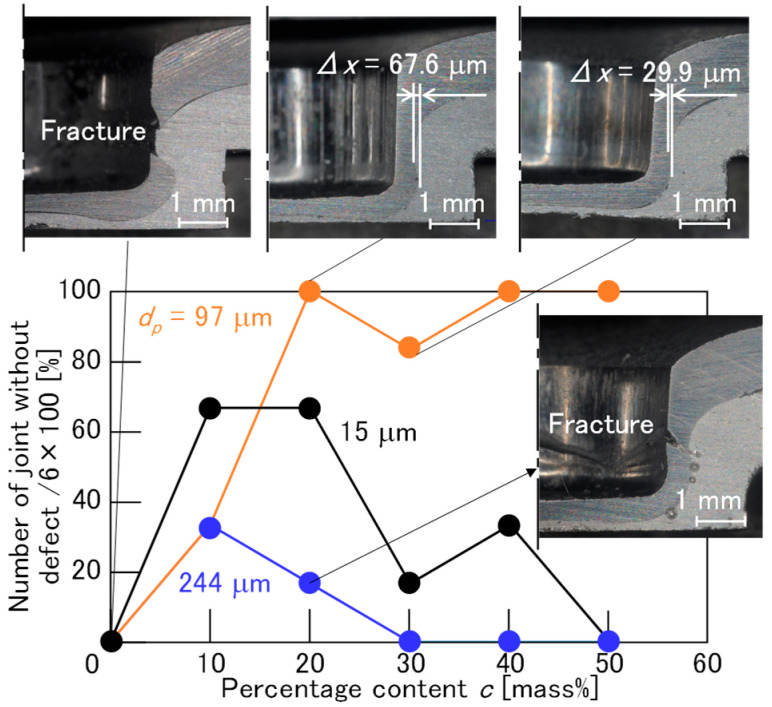
The effect of the percentage content of fine particles in the adhesive on joinability.

The effect of the percentage content of fine particles in the adhesive on joinability was evaluated using six joints for each condition, and the effect is shown in Figure 22. The percentage content *c* = 0 mass% represents the condition in the adhesive without fine particles. A fracture was prevented with an appropriate percentage content and particle diameter. Although the number of joints without defects was increased in the ranges of *c* = 10 mass% and 20 mass% in *d*_p_ = 15 μm and the ranges of *c* = 10 mass% in *d*_p_ = 244 μm, the optimal range for avoiding fracture was from *c* = 20 mass% to 50 mass% and *d*_p_ = 97 μm.

**Figure 23:**
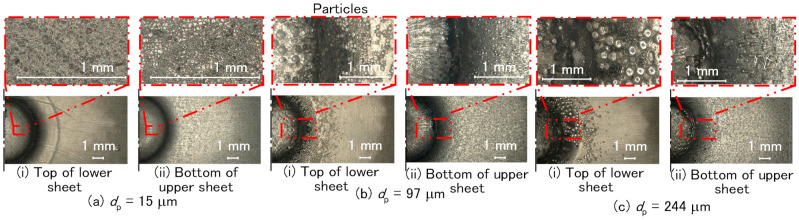
Fine particles at the interface of the upper and lower sheets. (**a**) *d*_p_ = 15 mm, (**b**) *d*_p_ = 97 mm, and (**c**) *d*_p_ = 244 mm.

The joined sheets before curing were cut, and then the adhesive was removed. The effect of fine particles in the adhesive at *c* = 20 mass% was investigated by observing the interface between the upper and lower sheets using the digital microscope. The fine particles at the interface of the upper and lower sheets are shown in Figure 23. In *d*_p_ = 15 μm, the fine indentations were observed on the top of the lower sheet and the bottom of the upper sheet. In *d*_p_ = 97 μm and *d*_p_ = 244 μm, not only the larger indentations in both surfaces but also the grooves caused by particle movement were observed on the bottom of the upper sheet. It seems that the material flow of the aluminum alloy sheet in *d*_p_ = 97 μm was reduced because the number of grooves in *d*_p_ = 97 μm was greater than that at *d*_p_ = 244 μm, although the width of the grooves in *d*_p_ = 244 μm was larger.

**Figure 24:**
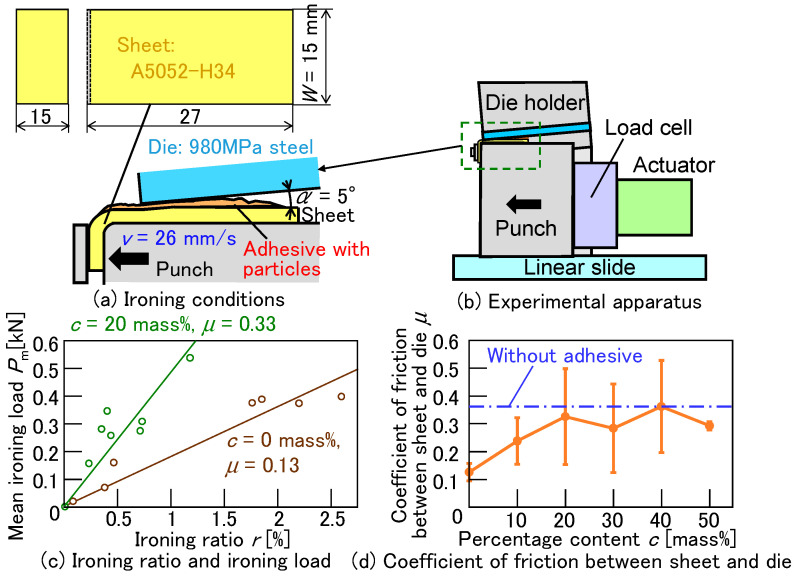
Ironing conditions and the coefficient of friction between the upper and lower sheets. (**a**) Ironing conditions, (**b**) experimental apparatus, (**c**) ironing ratio and ironing load, and (**d**) coefficient of friction between sheet and die.

The ironing conditions and the coefficient of friction between the upper and lower sheets are shown in Figure 24. To compare the frictional effects of the adhesive with fine particles, the coefficients of friction were measured by an ironing experiment. In ironing, the sheet thickness was reduced by the clearance between a punch and a die. The 980 MPa steel sheet and the A5052 sheet were used as the die and the sheet, respectively, with the adhesive containing fine particles applied between them, as shown in Figure 24a. The coefficient of friction *μ* in the plane strain condition [34] was calculated by

(3) $$\mu =\frac{1}{Cot\alpha }\left(\frac{P_{m}}{2Wt_{1}\tau \;ln\left(\frac{1}{1-r}\right)}-1\right).$$

where *t*_1_ (mm), *W* (mm), *α* (°), *τ* (MPa), *P*_m_(N), and *r* are the thickness of the ironed sheet, the sheet width, the die angle, the shear stress of the sheet, the ironing load, and the ironing ratio, respectively. The ironing ratio *r* is

(4) $$r=\frac{{t_{0}-t}_{1}}{t_{0}},$$

where *t*_0_ (mm) is the initial thickness of the sheet. Thicknesses *t*_0_ and *t*_1_ (mm) are measured by a micrometer (Mitutoyo Corporation, Kawasaki, Japan) before and after ironing. The die angle *α* = 5° was used. The shear stress of the sheet was assumed as

(5) $$\tau =\frac{\sigma_{B}}{\sqrt{3}}.$$

The ironing load was the mean load from the load measured by a load cell (UBFH-2KN, Unipulse Co., Saitama, Japan). The ironing ratio from 0 to 2.5% was ranged, set by the clearance between the punch and the die. The coefficients of friction were determined based on the ironing load and ironing ratio. The ironing speed of 26 mm/s was achieved using the actuator RCS3-RA20R (IAI Corp., Shizuoka, Japan). In Figure 24b, the relationship between the ironing load and the ironing ratio for *c* = 0 and 20 mass% is shown. The lines representing the coefficient of friction, calculated using equation (2), were inserted into the figure. Although the variations were observed, differences in the slope trends were evident. The effect of the percentage content of the fine particles on the coefficient of friction between the sheet and the die is shown in Figure 24c. The coefficients of friction when using adhesive with fine particles and without fine particles were approximately 0.13 and 0.36, respectively. Although significant variations were observed, the mean coefficient of friction increased from *c* = 0 mass% to *c* = 20 mass %, and then, the coefficient was almost constant over *c* = 20 mass%. The constant coefficient was high and close to the coefficient of friction without the adhesive. This indicates that the deformed shape of the sheets with fine ceramic particles corresponds to that shown in Figure 19 when the friction coefficient is μ = 0.35. The frictional effects of the adhesive with fine particles were observed in the ironing experiment, and then, the coefficient of friction increased approximately threefold when using the adhesive with the fine particles more than *c* = 20 mass%.

### 4.3. Experimental Results of Joint Strength

**Figure 25:**
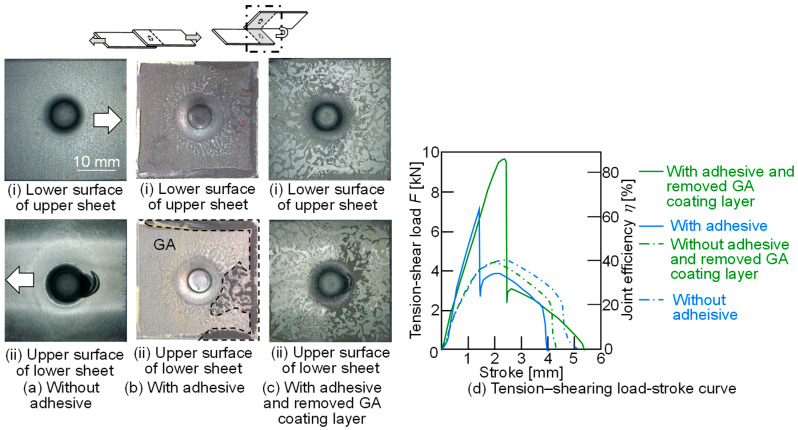
Tension–shear loads of joined sheets for *c* = 20 mass% and *L* = 50 mm. (**a**) Without adhesive, (**b**) with adhesive, (**c**) with adhesive and removed GA coating layer, and (**d**) tension-shear load.

The tension-shear loads of the joined sheets for *c* = 20 mass% and *L* = 50 mm are shown in Figure 25. As a comparison, a 980 MPa sheet with the GA coating layer removed was joined, and then, the tension-shear loads of the joints were measured with and without the adhesive. In the condition without the adhesive, separation occurred due to interlock pullout of the upper sheet. The maximum loads of the joints, with and without the GA coating layer, were nearly identical, approximately *η* = 40%. In the condition with the adhesive, peeling of both the adhesive layer and the GA coating layer was observed. The maximum load of the joint without the GA coating layer was higher than that of the joint with the GA coating layer. The upper 980 MPa steel and lower aluminum alloy sheets were successfully joined using clinching with the adhesive containing fine particles. The joint load was increased by the adhesive layer, although the strength of the GA coating layer appeared weaker than that of the adhesive layer.

**Figure 26:**
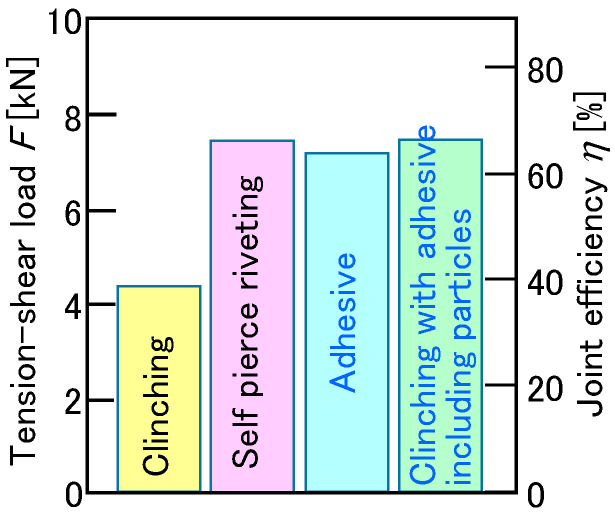
Tension–shear loads and joint efficiencies.

The tension-shear loads and the joint efficiencies are shown in Figure 26. As a comparison, the load of the joint by self-pierce riveting was measured. The joint load by mechanical clinching with the adhesive, including the fine particles, increased to the joint load by only adhesive and the joint by self-pierce riveting, which is *η* = approximately 65% from *η* = approximately 40% for mechanical clinching without the adhesive.

## 5. Conclusions

In this study, the clinch-bonding process was used to join 1.2 mm thick steel sheets and 1.5 mm thick aluminum alloy sheets. The effects of sheet combinations on the joined cross-sectional shapes and joint load were investigated. The tension-shear loads of the joints were summarized as joint efficiency and compared. Additionally, the effects of clinching conditions on joinability were examined for joining ultra-high-strength steel as the upper sheet and aluminum alloy as the lower sheet. Finally, these sheets were successfully joined using adhesive containing fine particles. Based on the results obtained, the following conclusions were drawn:

(1) In joining two sheets, including 780 MPa steel and A5052 sheets, the effect of the adhesive on the amount of interlock and the minimum thickness in the upper sheet was not significant; however, the effect of the sheet combination was observed. The maximum load with the adhesive exceeded 80% in joint efficiency. The maximum load for both 780 MPa sheets with the adhesive was approximately 65% in joint efficiency, which was the same as the joint efficiency of the adhesive-only joint.
(2) Although the steel and A5052 sheets were joined without defects in many conditions in different die shapes, punch speeds, and holding forces in the 590 MPa and 780 MPa steel sheets, the conditions without defects were limited in the 980 MPa steel and A5052 sheets. Not only the insufficient ductility of the steel sheet but also the material flow of the sheets by the frictional effect between the sheets with the adhesive was influenced.
(3) In joining the upper 980 MPa ultra-high-strength steel and lower aluminum alloy sheets using clinching with the adhesive, the fracture of the steel around the sidewall of the punch could be prevented by reducing the material flow of the steel and aluminum alloy sheets using adhesive containing fine particles to increase friction. The friction coefficient under optimal conditions with the adhesive containing fine particles was close to that without the adhesive.

To reduce the weight of automobiles, low-strength aluminum alloy sheets are combined with materials such as high-strength and ultra-high-strength steel sheets. In such sheet combinations, when the sheets are clinched together, the large difference in flow stress between them causes an increased flow of the low-strength material. Furthermore, when an adhesive such as epoxy is used, its high viscosity at the sheet interface promotes material flow. In this study, the material flow was controlled by increasing friction between the sheets. However, the study focuses only on specific thickness combinations. In practical applications, various thickness combinations will be required, and since there are many types of aluminum alloy and 980 MPa steel sheets, their deformation behaviors are expected to differ. Finite element simulation will be the main method for designing die shapes to control material flow, but developing friction control—as proposed in this study—is also important. For practical implementation, it will be necessary to evaluate not only joinability and joining load, but also the prediction of the gap between sheets after curing, corrosion resistance, and fatigue strength of joints over the service period.

## Figures and Tables

**Table 1:** Mechanical properties of sheets.

Sheet	Thickness *t* [mm]	Tensile Strength $\sigma_{B}$ [MPa]	Elongation [%]
780 MPa steel	1.20	799	19.2
A5052-H34	1.50	249	10.5

**Table 2:** Sheet combinations and clinching conditions for clinch-bonding (unit: mm).

Combination	Upper Sheet	Lower Sheet	*s* [mm]	*H* [mm]	*D* [mm]
Dissimilar	780 MPa steel	A5052-H34	3.6	1.5	8.5
	A5052-H34	780 MPa steel	4.0	1.7	8.0
Similar	A5052-H34	A5052-H34	3.9	1.7	8.75
	780 MPa steel	780 MPa steel	3.6	1.7	8.5

**Table 3:** Material properties of the steel sheets.

Sheet	Sheet	Thickness *t* [mm]	Tensile Strength $\sigma_{B}$ [MPa]	Elongation [%]
Upper	590 MPa steel	1.20	574	24.8
	780 MPa steel		799	19.2
	980 MPa steel		1035	13.9
Lower	A5052-H34	1.50	249	10.5

**Table 4:** Flow stresses of sheets.

Sheet	Thickness *t* [mm]	*K* [MPa]	*n* [-]
980 MPa steel	1.20	1406	0.13
A5052-H34	1.50	411	0.11

## Data Availability

The original contributions presented in the study are included in the article. Further inquiries can be directed to the corresponding author.
